# Supplementary material for: Structures of the lipid II flippase from the monoderm pathogen Staphylococcus aureus
Source: J Biol Chem. 2026 Sep 6;302(10):113516. doi: 10.1016/j.jbc.2026.113516 (PMC13661225; doi:10.1016/j.jbc.2026.113516)
Supplement: Supplementary file 1 [file mmc1.pdf]

Supporting information for

Structures of the lipid II flippase from the monoderm pathogen Staphylococcus aureus

Yancheng E. Li[1], Grace F. Baron[1], William M. Clemons, Jr.[1, 2, *]

[1]Division of Chemistry and Chemical Engineering, California Institute of Technology, Pasadena, California, USA

[2]Chan Zuckerberg Initiative, Redwood City, California, USA

[*]To whom correspondence may be addressed. **Email**: clemons@caltech.edu

This PDF file includes:

Figs. S1 to S9
Table S1
References (1-7)

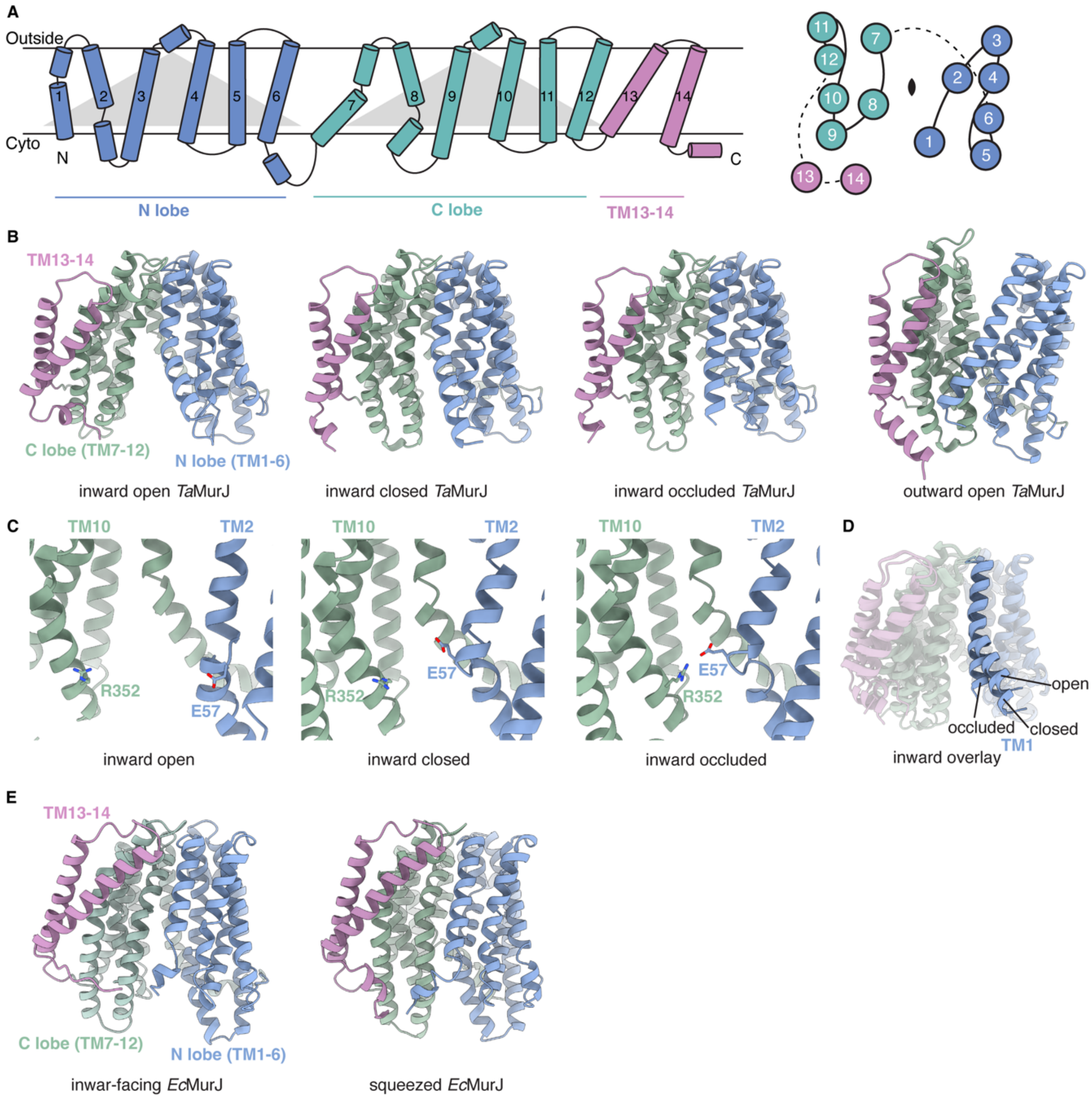

Figure S1. Architecture of MurJ. ***A,*** left, predicted topology of *Sa*MurJ showing the 14 numbered transmembrane helices organized into the N-lobe (TM1-6) (blue), C-lobe (TM7-12) (green), and TM13-14 (pink). Triangles indicate the symmetrical lobes. Right, top view representation of MurJ showing the arrangement of transmembrane helices surrounding the central cavity. TMs 1-12 are pseudo-C2-symmetric six-helix N- and C-lobes. ***B,*** crystal structures of *Thermosipho africanus* MurJ (*Ta*MurJ) (1) in distinct conformational states colored by domain architecture (PDB: 6NC7, 6NC6, 6NC8, 6NC9). ***C,*** close-up view of the thin gate between Glu57 in TM2 and Arg352 in TM10. ***D,*** structural overlay of inward structures of *Ta*MurJ highlighting the closure of TM1 at the membrane portal. ***E,*** crystal structures of *E. coli* MurJ (*Ec*MurJ) in an inward-facing state (2) (PDB: 6CC4) or a squeezed state (3) (PDB: 7WAG).

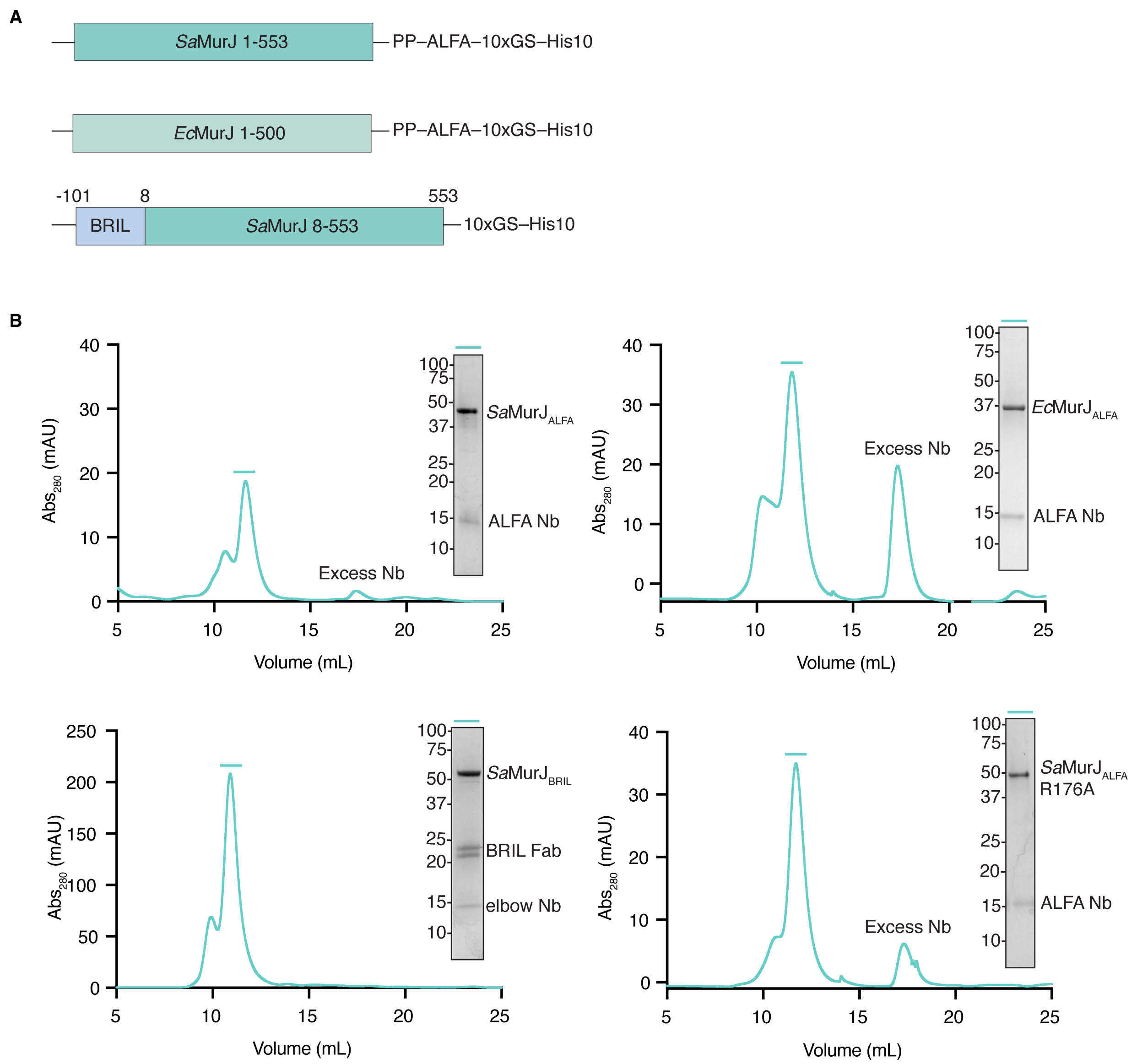

Figure S2. Construct design and purification of *Sa*MurJ and *Ec*MurJ proteins. *A,* schematic of expression constructs for *Sa*MurJ and *Ec*MurJ. *B,* representative size-exclusion chromatography profiles and SDS-PAGE analyses of purified *Sa*MurJ$_{ALFA}$-Nb$_{ALFA}$, *Sa*MurJ$_{BRIL}$-Fab-Nb$_{FAB}$, *Ec*MurJ$_{ALFA}$-Nb$_{ALFA}$ complexes.

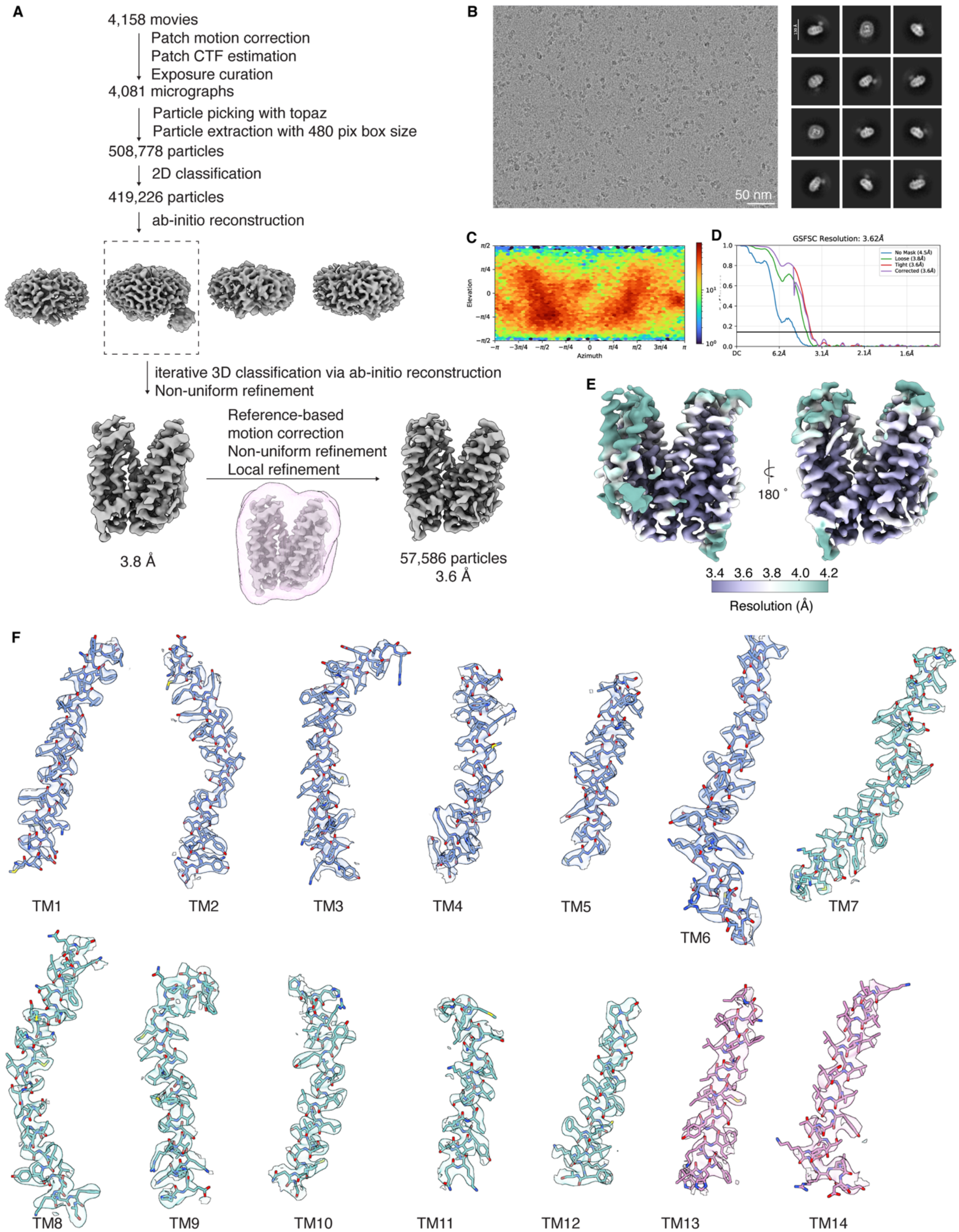

A
4,158 movies
Patch motion correction
Patch CTF estimation
Exposure curation
4,081 micrographs
Particle picking with topaz
Particle extraction with 480 pix box size
508,778 particles
2D classification
419,226 particles
ab-initio reconstruction
iterative 3D classification via ab-initio reconstruction
Non-uniform refinement
Reference-based
motion correction
Non-uniform refinement
Local refinement
3.8 Å
57,586 particles
3.6 Å
B
50 nm
C
Elevation
Azimuth
D
GSFSC Resolution: 3.62Å
No Mask (4.5Å)
Loose (3.8Å)
Tight (3.6Å)
Corrected (3.6Å)
E
180 °
3.4 3.6 3.8 4.0 4.2
Resolution (Å)
F
TM1
TM2
TM3
TM4
TM5
TM6
TM7
TM8
TM9
TM10
TM11
TM12
TM13
TM14

Figure S3. Cryo-EM structure determination of *Sa*MurJ in an outward-facing state. *A,* summary of cryo-EM data processing workflow to obtain the outward-facing structure of *Sa*MurJ. *B,* representative micrograph (left) and 2D class averages (right). *C,* angular distribution of particles of the final 3D reconstruction. *D,* Fourier shell correlation curves between two half maps. *E,* local resolution map of the final cryo-EM map colored by resolution from 3.4 Å (purple) to 4.2 Å (green). *F,* cryo-EM density of each TM helix of *Sa*MurJ. Map contour level = 0.059 in ChimeraX (4).

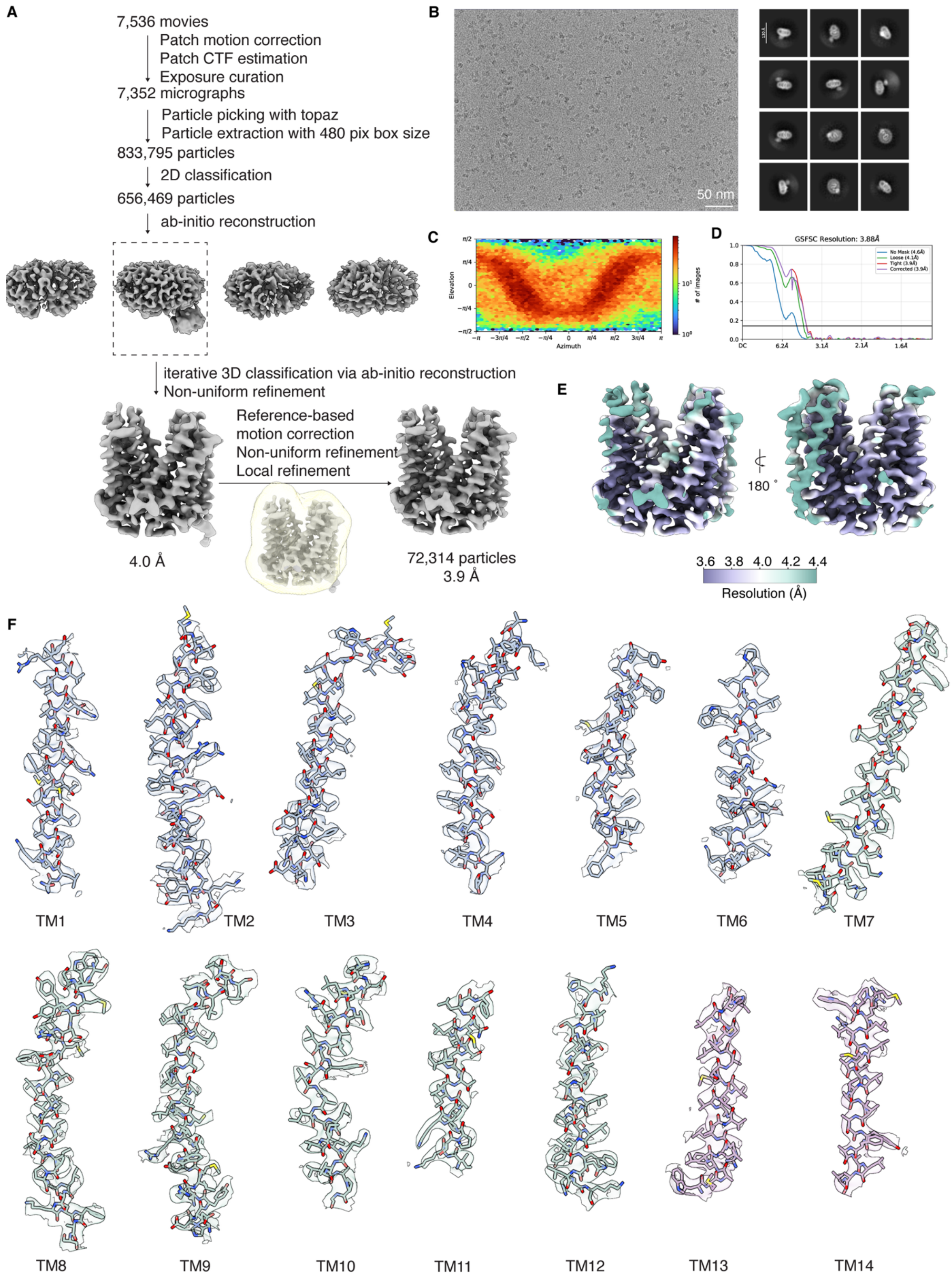
A
7,536 movies
Patch motion correction
Patch CTF estimation
Exposure curation
7,352 micrographs
Particle picking with topaz
Particle extraction with 480 pix box size
833,795 particles
2D classification
656,469 particles
ab-initio reconstruction
iterative 3D classification via ab-initio reconstruction
Non-uniform refinement
Reference-based
motion correction
Non-uniform refinement
Local refinement
4.0 Å
72,314 particles
3.9 Å
B
50 nm
C
Elevation
Azimuth
# of images
D
GSFSC Resolution: 3.88Å
No Mask (4.6Å)
Loose (4.1Å)
Tight (3.9Å)
Corrected (3.9Å)
DC
6.2Å
3.1Å
2.1Å
1.6Å
E
180 °
3.6
3.8
4.0
4.2
4.4
Resolution (Å)
F
TM1
TM2
TM3
TM4
TM5
TM6
TM7
TM8
TM9
TM10
TM11
TM12
TM13
TM14

Figure S4. Cryo-EM structure determination of *Ec*MurJ in an outward-facing state. *A,* summary of cryo-EM data processing workflow to obtain the outward-facing structure of *Ec*MurJ. *B,* representative micrograph (left) and 2D class averages (right). *C,* angular distribution of particles of the final 3D reconstruction. *D,* Fourier shell correlation curves between two half maps. *E,* local resolution map of the final cryo-EM map colored by resolution from 3.6 Å (purple) to 4.4 Å (green). *F,* cryo-EM density of each TM helix of *Ec*MurJ. Map contour level = 0.055 in ChimeraX (4).

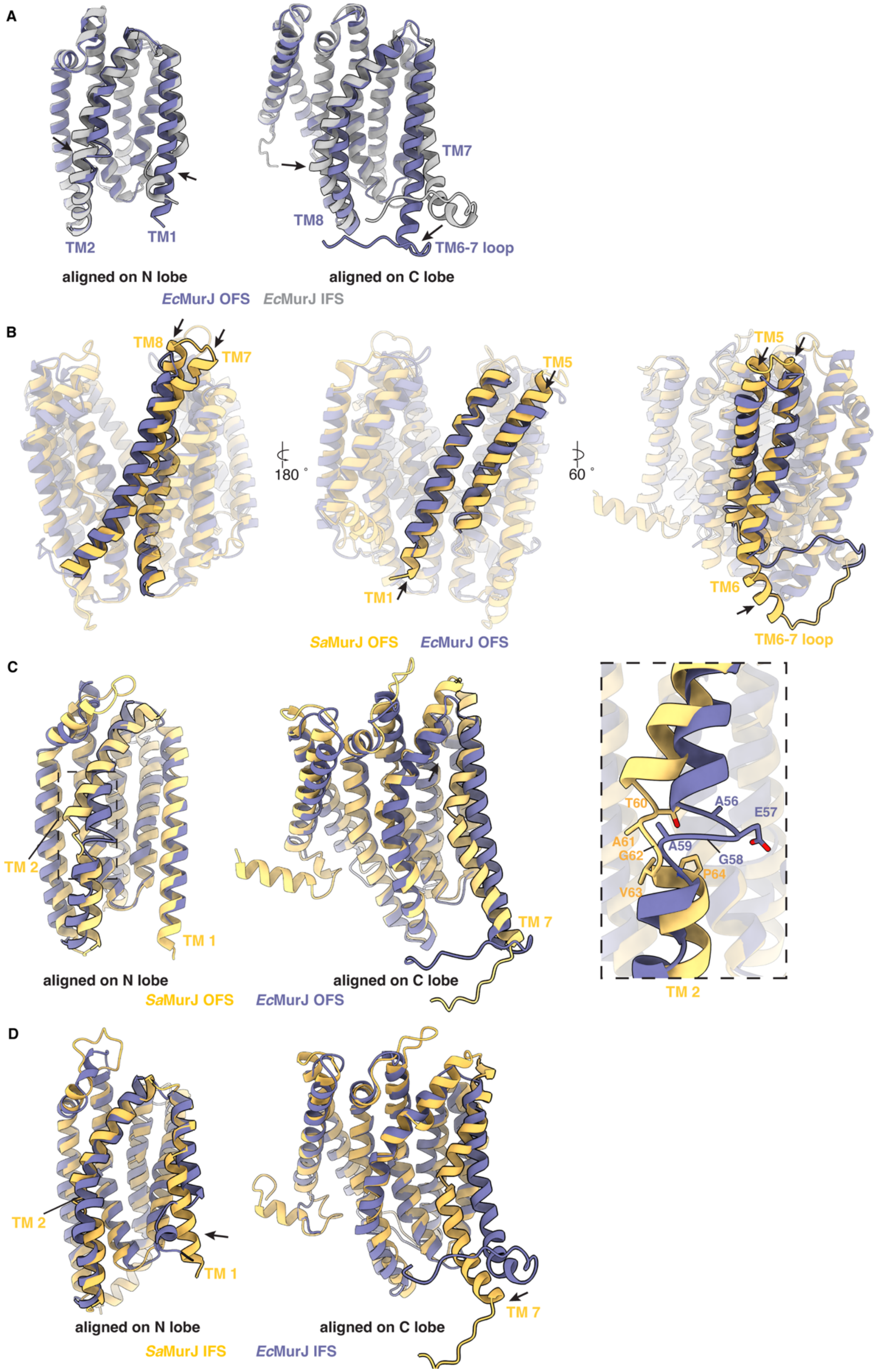
A
TM2
TM1
aligned on N lobe
TM7
TM8
TM6-7 loop
aligned on C lobe
EcMurJ OFS
EcMurJ IFS
B
TM8
TM7
180 °
TM5
TM1
60 °
TM5
TM6
TM6-7 loop
SaMurJ OFS
EcMurJ OFS
C
TM 2
TM 1
aligned on N lobe
TM 7
aligned on C lobe
SaMurJ OFS
EcMurJ OFS
T60
A56
E57
A61
G62
A59
G58
P64
V63
TM 2
D
TM 2
TM 1
aligned on N lobe
TM 7
aligned on C lobe
SaMurJ IFS
EcMurJ IFS

Figure S5. Structural comparison of *Sa*MurJ and *Ec*MurJ. ***A,*** structural overlay of the outward- and inward-facing *Ec*MurJ (PDB: 6CC4) aligned to either the N- or the C-lobe. ***B,*** structural overlay of outward-facing *Sa*MurJ and *Ec*MurJ highlighting the differences in TMs 1, 5–8, and TM6-7 loop. ***C,*** comparison of the outward-facing *Sa*MurJ and *Ec*MurJ aligned to either the N- or the C-lobe. Dashed box highlights structural and sequence differences in TM2. ***D,*** comparison of the inward-facing *Sa*MurJ and *Ec*MurJ (PDB: 6CC4) aligned to either the N- or the C-lobe.

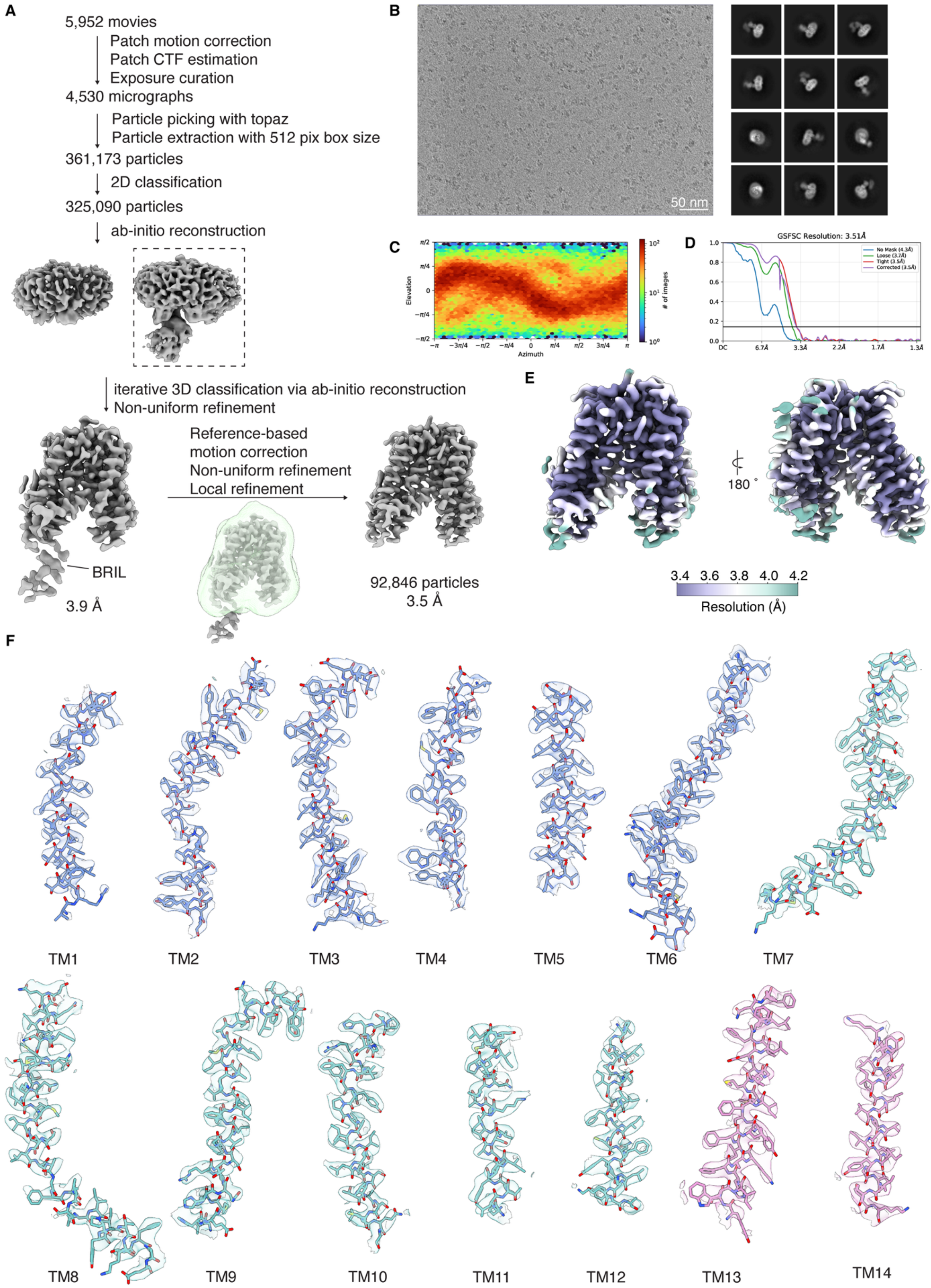
A
5,952 movies
Patch motion correction
Patch CTF estimation
Exposure curation
4,530 micrographs
Particle picking with topaz
Particle extraction with 512 pix box size
361,173 particles
2D classification
325,090 particles
ab-initio reconstruction
iterative 3D classification via ab-initio reconstruction
Non-uniform refinement
Reference-based
motion correction
Non-uniform refinement
Local refinement
BRIL
3.9 Å
92,846 particles
3.5 Å
B
50 nm
C
Elevation
Azimuth
# of images
D
GSFSC Resolution: 3.51Å
No Mask (4.3Å)
Loose (3.7Å)
Tight (3.5Å)
Corrected (3.5Å)
E
180 °
3.4 3.6 3.8 4.0 4.2
Resolution (Å)
F
TM1
TM2
TM3
TM4
TM5
TM6
TM7
TM8
TM9
TM10
TM11
TM12
TM13
TM14

Figure S6. Cryo-EM structure determination of *Sa*MurJ in an inward-facing state. *A,* summary of cryo-EM data processing workflow to obtain the inward-facing structure of *Sa*MurJ. *B,* representative micrograph (left) and 2D class averages (right). *C,* angular distribution of particles of the final 3D reconstruction. *D,* Fourier shell correlation curves between two half maps. *E,* local resolution map of the final cryo-EM map colored by resolution from 3.4 Å (purple) to 4.2 Å (green). *F,* cryo-EM density of each TM helix of *Sa*MurJ. Map contour level = 0.061 in ChimeraX (4).

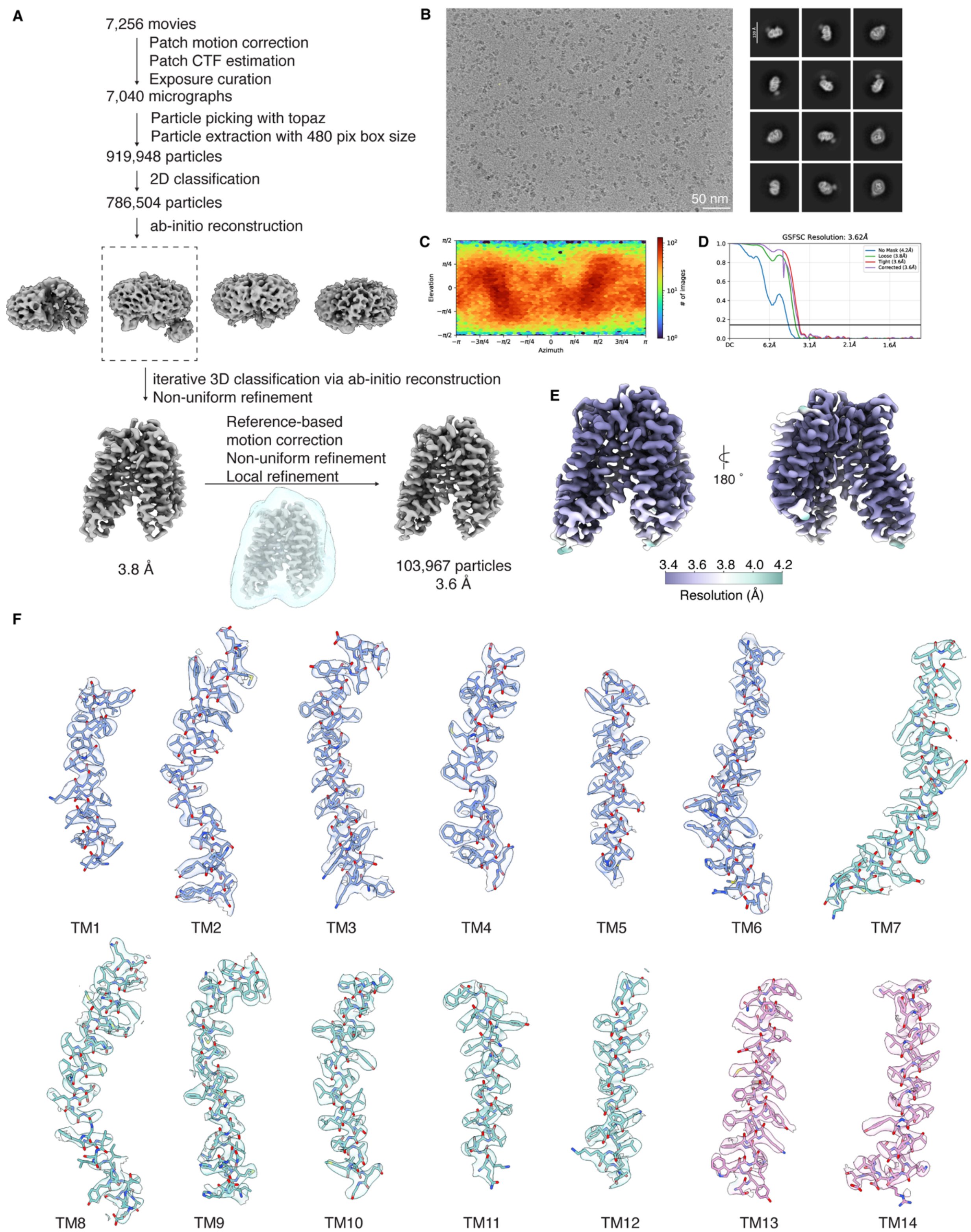
A
7,256 movies
Patch motion correction
Patch CTF estimation
Exposure curation
7,040 micrographs
Particle picking with topaz
Particle extraction with 480 pix box size
919,948 particles
2D classification
786,504 particles
ab-initio reconstruction
iterative 3D classification via ab-initio reconstruction
Non-uniform refinement
Reference-based
motion correction
Non-uniform refinement
Local refinement
3.8 Å
103,967 particles
3.6 Å
B
50 nm
C
Elevation
Azimuth
# of images
D
GSFSC Resolution: 3.62Å
No Mask (4.2Å)
Loose (3.8Å)
Tight (3.6Å)
Corrected (3.6Å)
E
180 °
3.4 3.6 3.8 4.0 4.2
Resolution (Å)
F
TM1
TM2
TM3
TM4
TM5
TM6
TM7
TM8
TM9
TM10
TM11
TM12
TM13
TM14

Figure S7. Cryo-EM structure determination of *Sa*MurJ$_{ALFA}$ R176A variant. *A,* summary of cryo-EM data processing workflow to obtain the structure of *Sa*MurJ R176A variant. *B,* representative micrograph (left) and 2D class averages (right). *C,* angular distribution of particles of the final 3D reconstruction. *D,* Fourier shell correlation curves between two half maps. *E,* local resolution map of the final cryo-EM map colored by resolution from 3.4 Å (purple) to 4.2 Å (green). *F,* cryo-EM density of each TM helix of *Sa*MurJ R176A variant. Map contour level = 0.08 in ChimeraX (4).

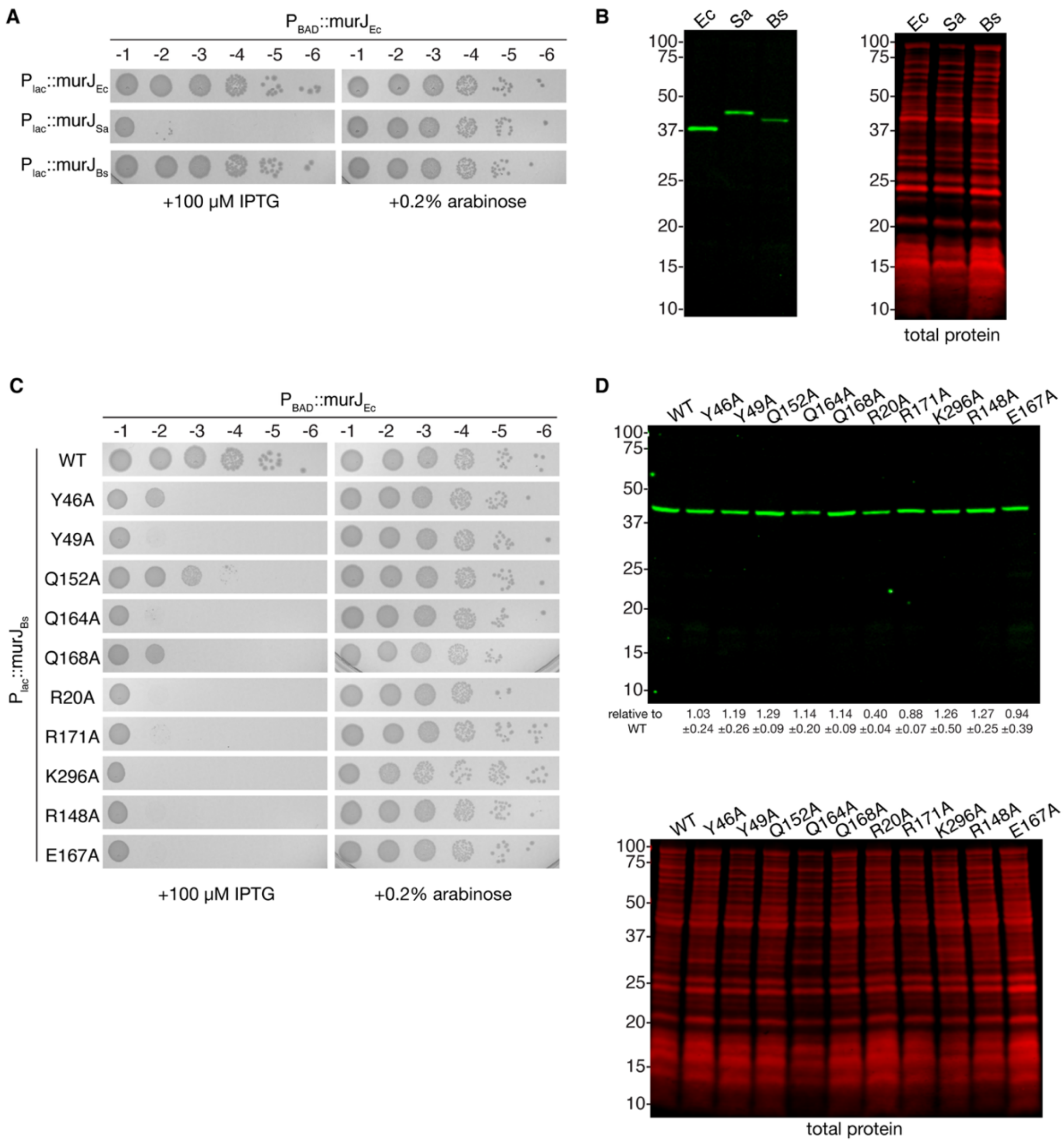

Figure S8. Complementation by MurJ homologs and variants. *A,* complementation by MurJ homologs in a MurJ-depletion *E. coli* strain (5). The chromosomal $murJ_{Ec}$ is controlled by an arabinose-inducible promoter. MurJ homologs are expressed from an IPTG-inducible P_{lac} promoter. *B,* representative western blots of the membrane fractions from *E. coli* cells expressing different MurJ homologs. Molecular weight markers are indicated for all western blots. *C,* complementation by *Bs*MurJ mutants. *D,* representative western blots of the membrane fractions from *E. coli* cells expressing *Bs*MurJ mutants. Values below each lane indicate the protein level normalized to total protein staining relative to wild-type, shown as mean ± s.d. from three biological replicates.

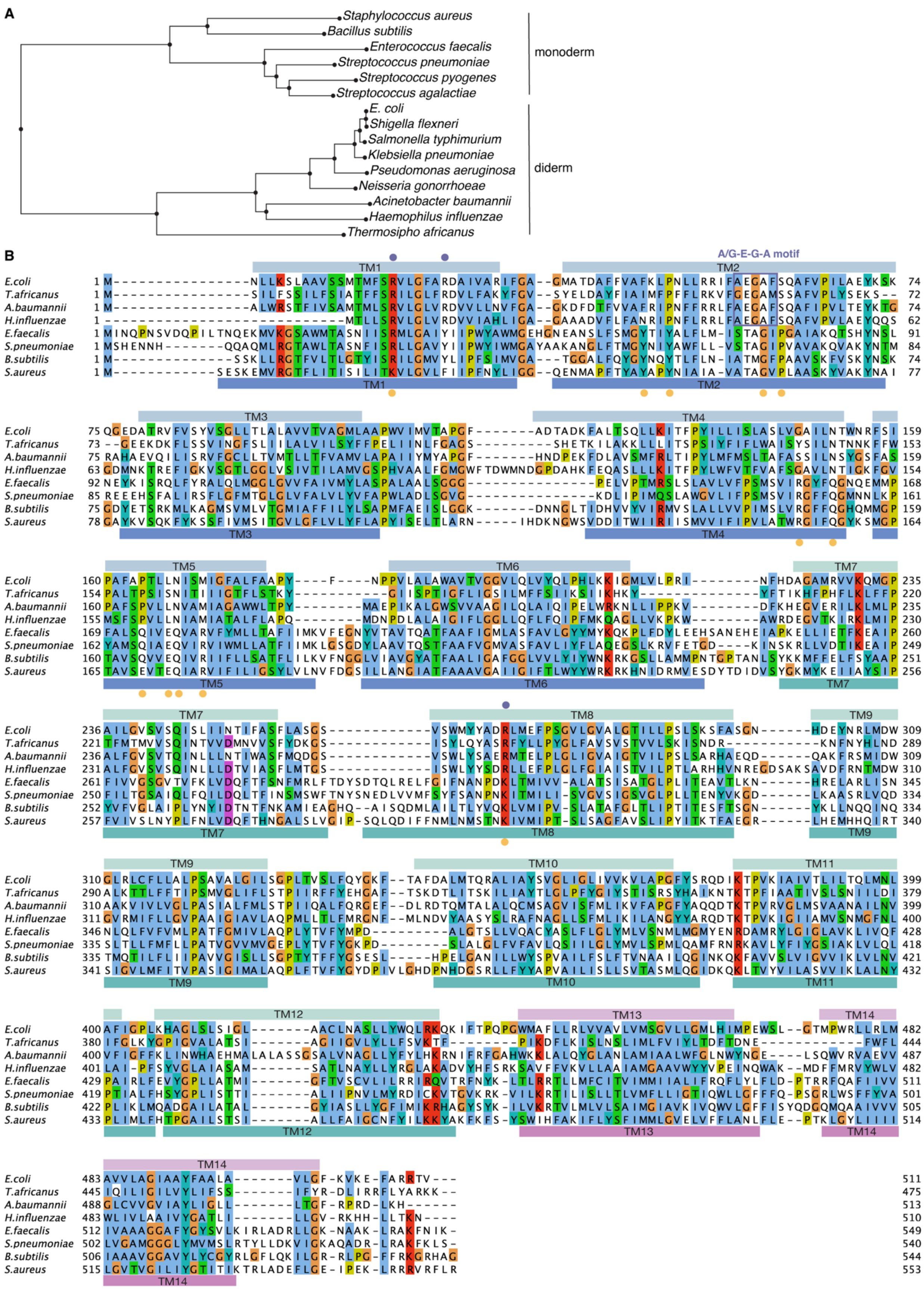
A
Staphylococcus aureus
Bacillus subtilis
Enterococcus faecalis
Streptococcus pneumoniae
Streptococcus pyogenes
Streptococcus agalactiae
E. coli
Shigella flexneri
Salmonella typhimurium
Klebsiella pneumoniae
Pseudomonas aeruginosa
Neisseria gonorrhoeae
Acinetobacter baumannii
Haemophilus influenzae
Thermosipho africanus
monoderm
diderm
B
A/G-E-G-A motif
TM1
TM2
TM3
TM4
TM5
TM6
TM7
TM8
TM9
TM10
TM11
TM12
TM13
TM14
E.coli
T.africanus
A.baumannii
H.influenzae
E.faecalis
S.pneumoniae
B.subtilis
S.aureus

Figure S9. Sequence alignment of representative MurJ homologs from priority bacterial pathogens. ***A,*** phylogenetic tree of representative monoderm and diderm MurJ homologs from the WHO Bacterial Priority Pathogens List 2024 (6). *Thermosipho africanus* is included for completeness. ***B,*** structure-based multiple sequence alignment using PROMALS3D (7) with ClustalW coloring for residues. Species were selected from the phylogenetic tree in *A*. MurJ homologs are (UniProt IDs in parentheses): *Escherichia coli* (P0AF16), *Acinetobacter baumannii* (D0CEW3), *Haemophilus influenzae* (P44958), *Thermosipho africanus* (B7IE18), *Staphylococcus aureus* (Q2FXH6), *Enterococcus faecalis* (Q838A3), *Streptococcus pneumoniae* (Q8DP32), and *Bacillus subtilis* (O34674). Secondary structures are shown above the sequence for *Ec*MurJ and below the sequence for *Sa*MurJ colored as in Fig. 1. Functionally important residues are highlighted by circles (purple: *Ec*MurJ, yellow: *Sa*MurJ), including conserved residues implicated in lipid II coordination and conformational rearrangements.

Table S1. Cryo-EM data collection, refinement, and validation statistics

	*Sa*MurJ outward (EMD-76175) (PDB 11XX)	*Ec*MurJ outward (EMD-76176) (PDB 11XY)	*Sa*MurJ inward (EMD-76177) (PDB 11XZ)	*Sa*MurJ R176A (EMD-76178) (PDB 11YA)
**Data collection and processing**				
Microscope	FEI Titan Krios	FEI Titan Krios	FEI Titan Krios	FEI Titan Krios
Magnification	130,000	130,000	130,000	130,000
Voltage (kV)	300	300	300	300
Electron exposure (e–/Å^2)	70	70	70	70
Defocus range (μm)	1.0 to 3.0	1.0 to 3.0	1.0 to 3.0	1.0 to 3.0
Pixel size (Å)	0.649	0.649	0.649	0.649
Symmetry imposed	C1	C1	C1	C1
Initial particle images (no.)	508,778	833,795	361,173	919,948
Final particle images (no.)	57,586	72,314	92,846	103,967
Map resolution (Å)	3.6	3.9	3.5	3.6
FSC threshold	0.143	0.143	0.143	0.143
Map sharpening *B* factor (Å^2)	−127.1	−160.4	−122.3	−142.9
**Refinement**				
Initial model used (PDB code)	SaMurJ AF model	9NU4	SaMurJ AF model	SaMurJ AF model
Model resolution (Å)	3.8	4.0	3.7	3.8
FSC threshold	0.5	0.5	0.5	0.5
Model composition				
Non-hydrogen atoms	4382	3792	4327	4303
Protein residues	553	499	546	544
Ligands	0	0	0	0
B factors (Å^2)				
Protein	78.68	62.41	54.32	64.08
Ligand	---	---	---	---
R.m.s. deviations				
Bond lengths (Å)	0.006	0.006	0.008	0.005
Bond angles (°)	0.697	0.819	0.852	0.699
Validation				
MolProbity score	1.38	1.43	1.40	1.28
Clashscore	6.92	7.81	7.24	5.23
Poor rotamers (%)	0	0	0	0
Ramachandran plot				
Favored (%)	98.19	98.59	98.53	98.15
Allowed (%)	1.81	1.41	1.47	1.85
Disallowed (%)	0	0	0	0

SI References

1. Kuk, A. C. Y., Hao, A., Guan, Z., and Lee, S.-Y. (2019) Visualizing conformation transitions of the Lipid II flippase MurJ. *Nat. Commun.* 10, 1736
2. Zheng, S., Sham, L.-T., Rubino, F. A., Brock, K. P., Robins, W. P., Mekalanos, J. J., Marks, D. S., Bernhardt, T. G., and Kruse, A. C. (2018) Structure and mutagenic analysis of the lipid II flippase MurJ from *Escherichia coli*. *Proc. Natl. Acad. Sci.* 115, 6709-6714
3. Kohga, H., Mori, T., Tanaka, Y., Yoshikaie, K., Taniguchi, K., Fujimoto, K., Fritz, L., Schneider, T., and Tsukazaki, T. (2022) Crystal structure of the lipid flippase MurJ in a "squeezed" form distinct from its inward- and outward-facing forms. *Structure.* 30, 1088-1097
4. Goddard, T. D., Huang, C. C., Meng, E. C., Pettersen, E. F., Couch, G. S., Morris, J. H., and Ferrin, T. E. (2018) UCSF ChimeraX: Meeting modern challenges in visualization and analysis. *Protein Sci.* 27, 14–25
5. Sham, L.-T., Butler, E. K., Lebar, M. D., Kahne, D., Bernhardt, T. G., and Ruiz, N. (2014) MurJ is the flippase of lipid-linked precursors for peptidoglycan biogenesis. *Science.* 345, 220–222
6. WHO Bacterial Priority Pathogens List 2024: Bacterial Pathogens of Public Health Importance, to Guide Research, Development, and Strategies to Prevent and Control Antimicrobial Resistance (2024) 1st ed, World Health Organization, Geneva
7. Pei, J., Kim, B.-H., and Grishin, N. V. (2008) PROMALS3D: a tool for multiple protein sequence and structure alignments. *Nucleic Acids Res.* 36, 2295–2300
